# Exome sequencing identifies a likely causative variant in 53% of families with ciliopathy-related features on renal ultrasound after excluding *NPHP1* deletions

**DOI:** 10.1016/j.gendis.2023.101111

**Published:** 2023-09-15

**Authors:** Konstantin Deutsch, Verena Klämbt, Thomas M. Kitzler, Tilman Jobst-Schwan, Ronen Schneider, Florian Buerger, Steve Seltzsam, Sherif El Desoky, Jameela A. Kari, Farkhanda Hafeez, Maria Szczepańska, Loai A. Eid, Hazem S. Awad, Muna Al-Saffar, Neveen A. Soliman, Velibor Tasic, Camille Nicolas-Frank, Kirollos Yousef, Luca M. Schierbaum, Sophia Schneider, Abdul Halawi, Izzeldin Elmubarak, Katharina Lemberg, Shirlee Shril, Shrikant M. Mane, Nancy Rodig, Friedhelm Hildebrandt

**Affiliations:** a Division of Nephrology, Department of Pediatrics, Boston Children's Hospital, Harvard Medical School, Boston, MA 02215, USA; b Department of Pediatric Gastroenterology, Nephrology and Metabolic Diseases, Charité Universitätsmedizin Berlin, Berlin 13353, Germany; c Berlin Institute of Health at Charité – Universitätsmedizin Berlin, BIH Biomedical Innovation Academy, BIH Charité Clinician Scientist Program, Berlin 10178, Germany; d Department of Nephrology and Hypertension, Friedrich-Alexander-Universität Erlangen-Nürnberg, Erlangen 91054, Germany; e Department of Pediatrics, Faculty of Medicine King Abdulaziz University, Pediatric Nephrology Center of Excellence, King Abdulaziz University Hospital, Jeddah 21589, Saudi Arabia; f Department of Pediatric Nephrology, The Children's Hospital and Institute of Child Health, Lahore 54000, Pakistan; g Department of Pediatrics, Faculty of Medical Sciences in Zabrze, Medical University of Silesia in Katowice, Katowice 40-752, Poland; h Dubai Hospital and Al-Jalila Children's Specialty Hospital, Kidney Center of Excellence, Dubai 4545, United Arab Emirates; i Department of Genetics and Genomics, UAE University, Abu Dhabi 15551, United Arab Emirates; j Department of Pediatrics, Division of Genetics and Genomics, Boston Children's Hospital, Harvard Medical School, Boston, MA 02215, USA; k Department of Pediatrics, Center of Pediatric Nephrology and Transplantation, Kasr Al Ainy School of Medicine, Cairo University, Cairo 11562, Egypt; l Egypt Center for Research and Regenerative Medicine (ECRRM), Cairo 11511, Egypt; m Medical Faculty Skopje, University Children's Hospital, Skopje 1000, North Macedonia; n Department of Genetics, Yale University School of Medicine, New Haven, CT 06520, USA

Nephronophthisis-related ciliopathies (NPHP-RC) represent one of the most common causes of chronic kidney disease in the first three decades of life and are characterized by a broad genetic and clinical heterogeneity.^1^ To date, more than 90 genes have been identified that cause autosomal-recessive NPHP-RC if mutated, accounting for up to 60% of cases.^1^ Among these, homozygous deletions of *NPHP1* are the most common cause. Ciliopathy genes localize to primary cilia, basal bodies, or the centrosome and lead to a primary ciliary disruption if mutated, thereby causing a broad phenotypical spectrum.^1^ Patients that suffer from NPHP-RC can either have an isolated renal phenotype, such as polyuria, polydipsia, decreased urinary concentration ability, and secondary enuresis due to loss of tubular function, or present with extrarenal symptoms including retinal degeneration, cerebellar vermis hypoplasia, or hepatic fibrosis. Patients are often diagnosed in early adolescence, reaching end-stage renal disease before 25 years of age, but early onset and rapidly progressive forms of NPHP-RC also exist.^1^ Renal ultrasound indicates kidneys of normal or reduced renal length with increased echogenicity and corticomedullary cysts.

Due to reduced cost and widely available genomic sequencing, the detection rate of genetic causes of NPHP-RC has increased. To investigate the clinical and genetic findings of a worldwide cohort of 102 families with a clinical diagnosis of NPHP-RC, we performed exome sequencing and homozygosity mapping according to our inclusion criteria (see *Supplementary Methods*) after obtaining study approval, informed consent, and a clinical questionnaire.

To assess for likely causative variants in known monogenic causes of renal disease, we evaluated for variants in 96 genes known as monogenic causes of NPHP-RC if mutated (Table S1) and in 84 genes known to be causative of kidney disease mimicking an NPHP-RC phenotype (“phenocopies”) (Table S2; *Supplementary Methods*). We established a monogenic diagnosis in 54 of 102 (53%) families that had a clinical diagnosis of NPHP-RC (Fig. 1 and Table S3, 4). Of these 54 families, 45 (83%) harbored variants in one of the 96 known NPHP-RC genes (Fig. 1 and Table S3). The most frequently identified gene was *PKHD1* (17/54 families, 31%), followed by *CEP290* (5/54 families, 9%) (Table S3). In 9 families (17%) that were clinically diagnosed with NPHP-RC, we were able to identify a causative variant in a gene that leads to a kidney disease phenotype mimicking a ciliopathy (“phenocopies”) (Fig. 1 and Table S4). Variants were identified in genes causative of congenital anomalies of the kidney and the urinary tract in 4 families (*PAX2*, *HNF1B*), and 2 families carried deleterious variants in genes causative of Alport syndrome (*COL4A3*, *COL4A5*) or hyperoxaluria (*AGXT*) respectively. In one family with a clinical diagnosis of NPHP-RC, we identified a deleterious variant in *RMND1* leading to combined oxidative phosphorylation deficiency (OMIM# 614922) (Table S2).

**Figure 1:**
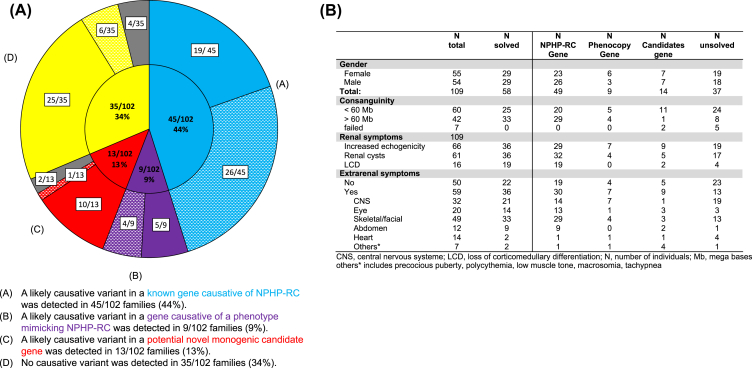
Results of exome sequencing analysis of 102 families with NPHP-RC. **(A)** Exome sequencing data of 109 affected individuals of 102 families with NPHP-RC were analyzed for likely causative variants in known NPHP-RC genes (96 genes, Table S1) or known phenocopy genes (84 genes, Table S2). The inner circle of the pie chart summarizes the main results of exome sequencing analysis indicating the number of families solved for a known NPHP-RC gene [A: blue, 45 of 102 families (44%)] or for a phenocopy gene [B: purple, 9 of 102 families (9%)]. Additionally, it shows the number of families in whom we identified a potential novel cause of NPHP-RC [C: red, 13 of 102 families (13%)], and the number of families in which we did not detect a likely causative variant and where thus declared as unsolved [D: yellow, 35 of 102 families (34%)]. The outer circle of the pie chart divides each group into families with consanguinity lower than 60 megabase pairs (solid color) or greater than 60 megabase pairs (hatched color). The grey parts of the pie chart indicate the number of families in which homozygosity mapping has failed. **(B)** Clinical characteristics of 109 individuals from 102 families with a presumptive diagnosis of NPHP-RC, defined by increased echogenicity, loss of corticomedullary differentiation, and/or ≥ 2 cysts on renal ultrasound.

We performed homozygosity mapping on all 102 families in whom exome sequencing was performed as previously described.^2^ 37 families had a homozygosity by descent of more than 60 megabase pairs. In this group, we were able to detect a likely causative monogenic variant in 30 of 37 (81%) families (Fig. 1). 59 families had a homozygosity by descent of less than 60 megabase pairs. Here, we detected a likely causative variant in only 24 of 59 (41%) families (Fig. 1). Of note, we identified a higher number of potential novel monogenic candidates for NPHP-RC in patients with low homozygosity (10/59 patients), compared with those with high homozygosity (1/37) (Fig. 1). Homozygosity mapping failed for 6 of the 102 families (Fig. 1).

Importantly, before performing exome sequencing, we excluded patients with *NPHP1* deletions. Previous studies that have focused on familial or consanguineous NPHP-RC cases have included patients with *NPHP1* deletions,^3^ or analyzed variants in only a subset of NPHP-RC genes.^4^ Thus, different genetic “solve rates” have been published explaining up to 64% of the cases.^3^^,^^5^ We recently demonstrated that 63% of 79 familial or consanguineous NPHP-RC families, in which an *NPHP1* deletion was excluded, harbored a likely causative variant in a monogenic kidney disease gene.^5^ Koenig et al found a monogenic cause in 97 of 152 (64%) NPHP-RC patients of a non-consanguineous European cohort.^3^ However, after excluding these cases, most cases were explained by an *NPHP1* deletion (60/152; 39%), equivalent to a genetic solve rate of 40%.^3^

In the last analysis step, we excluded all previously solved families to assess potential novel monogenic candidate genes causative of NPHP-RC. Variants in the remaining 45/102 families (44%) (Fig. 1) were evaluated using deleteriousness prediction software, allele frequency analysis in healthy control populations, conservation amongst phylogeny, and tissue expression datasets (see *Supplementary Methods*). We identified 13 potential novel candidate genes (*GLOD5*, *MEAK7*, *NHSL2*, *GPR63*, *NTN5*, *TRPM4*, *ZNF679*, *GPRC6A*, *TNS1*, *IQCA1*, *TFCP2L1*, *DEGS1*, and *RHPN1)* in 13 of 102 (13%) families (Fig. 1 and Table S5). However, each gene is so far only reported in 1 family. Identification of additional families with variants in one of the potential novel genes plus functional data is required to accept one of the novel NPHP-RC genes. In 35 (34%) families, we were unable to establish a molecular diagnosis. Here, we did not identify any deleterious biallelic variants in known or potential novel NPHP-RC genes (Fig. 1 and Table S6).

NPHP-RC patients can present with extrarenal manifestations in addition to their renal phenotype. We found that involvement of one or more organ systems besides the renal phenotype increased the likelihood of a molecular diagnosis in a known renal disease gene (37/59 patients, 63%) as well as the identification of a potential novel monogenic cause of NPHP-RC (9/59 patients, 15%) compared with the absence of extrarenal symptoms (24/50 patients (48%) and 5/50 patients (10%), respectively) (Fig. 1).

In summary, we performed exome sequencing combined with homozygosity mapping in 109 individuals from 102 families with a presumptive clinical diagnosis of NPHP-RC. We detected a monogenic variant likely causative of the patient's phenotype in 54 of 102 (53%) families. In addition, we identified 13 potential novel NPHP-RC candidate genes. Our study emphasizes the clinical relevance of performing exome sequencing in patients with a presumptive diagnosis of NPHP-RC. Due to the phenotypic heterogeneity of renal ciliopathies and diseases mimicking such phenotypes, it is imperative to establish a timely molecular diagnosis in patients with an NPHP-RC phenotype.

## Ethics declaration

This study was approved by the institutional review boards of the University of Freiburg, the University of Michigan, and Boston Children's Hospital. Informed consent for participation was given by the patients or their legal guardians before collecting clinical data and pedigree information (www.renalgenes.org). F. Hildebrandt is a cofounder and S.A.B. member of Goldfinch Biopharma Inc.

## Conflict of interests

F. Hildebrandt is a cofounder and S.A.B. member of Goldfinch Biopharma Inc. The other authors have no competing interests to declare.

## Funding

The Deutsche Forschungsgemeinschaft funded V.K. (No. 403877094), S.Se (No. 442070894) and T.J.S. (No. 281319475). V.K. was also funded by the Else-Kröner Fresenius Stiftung (Memorial Grant), the BIH Charité Clinician Scientist Program by the Charité – Universitätsmedizin Berlin, and the Berlin Institute of Health at Charité. S.Se. was supported by the Deutsche Forschungsgemeinschaft (German Research Foundation). T.M.K. was also supported by a Post-Doctoral Fellowship award from the KRESCENT Program, a national kidney research training partnership of the Kidney Foundation of Canada, the Canadian Society of Nephrology, and the Canadian Institutes of Health Research. T.J.-S. received funding from the IZKF Erlangen (J70) and the Eva Luise und Horst Köhler Stiftung/Else Kröner-Fresenius Stiftung (RECORD program).

## Appendix A Supplementary data

Multimedia component 1 Detailed materials and methods

Multimedia component 2 Monogenic causes of NPHP-RC

Multimedia component 3 Genes mimicking NPHP-RC phenotypes

Multimedia component 4 Families solved for NPHP-RC

Multimedia component 5 Families solved for phenocopies

Multimedia component 6 Families solved for potential novel NPHP-RC genes

Multimedia component 7 Unsolved families
