# Supplementary material for: Exome sequencing identifies a likely causative variant in 53% of families with ciliopathy-related features on renal ultrasound after excluding NPHP1 deletions
Source: Genes Dis. 2023 Sep 15;11(5):101111. doi: 10.1016/j.gendis.2023.101111 (PMC11167256; doi:10.1016/j.gendis.2023.101111)
Supplement: Multimedia component 1 [file mmc1.docx]

**DETAILED MATERIALS AND METHODS**

**Human subjects:** This study was approved by the institutional review boards of the University of Freiburg, University of Michigan and Boston Children’s Hospital. Informed consent for participation was given by the patients or their legal guardians before collecting clinical data and pedigree information (www.renalgenes.org). DNA samples were obtained between 09/2003 and 08/2019. None of the patients have previously been reported.

**Inclusion criteria:** Individuals included in this study were required to exhibit one or more ultrasonographic hallmark signs of cystic kidney disease and NPHP-RC. Inclusion criteria were increased echogenicity, loss of corticomedullary differentiation and/or identification of two or more renal cysts on kidney ultrasound examination, along with an age of onset of under 25 years of age. Prior to send-out for whole exome sequencing, patients were evaluated for deletions in *NPHP1*, and those with homozygous deletions in *NPHP1* were excluded from WES. Ultimately, we included 109 patients from 102 families and submitted these for ES.

**Variant calling:** Genomic DNA was isolated from whole blood or saliva samples according to local protocols. The Yale Center for Mendelian Genomics performed Whole Exome Sequencing on Agilent SureSelect™ human exome capture arrays (Agilent Technologies, California, USA), following next generation sequencing on an Illumina HiSeq™ sequencing platform (Illumina, California, USA). The resulting reads were then aligned to the human reference genome assembly (NCBI build GRCh37/hg19) with the CLC Biomedical Genomics Workbench™ (version 5.0.1) software (QIAGEN Aarhus A/S, Aarhus, Denmark). The average depth of coverage in our WES analysis was 30x. To ensure coverage of variants in NPHP1, we additionally ran separate coverage analysis for NPHP1. Variants with a minor allele frequency of >1% in the dbSNP database (version 147) were considered unlikely to be deleterious and excluded from analysis accordingly, as previously described. Additionally, we excluded synonymous variants and those in noncoding regions outside of splice sites. Non-synonymous variants and those in splice sites were analyzed for pathogenicity.

**Linkage analysis** Genome-wide homozygosity mapping was generated as previously described^2^ to identify regions of homozygosity as described. This method was performend in patients that were unsolved for a known NPHP-RC or phenocopy gene. For potential novel gene evaluation, first regions with high homozygosity were analyzed.

**Variant analysis** We examined WES data for potentially deleterious variants in any of the 96 canonical NPHP-RC genes (**Suppl. Table 1**) and in any of 84 genes known to mimic an NPHP-RC phenotype (**Suppl. Table 2**). If variants were previously reported as pathogenic in individuals with a matching phenotype, they were considered as likely deleterious. Previously unreported novel variants and variants in genes previously unpublished as potential novel monogenic causes of NPHP-RC were assessed for deleteriousness using the in-silico prediction programs Alamut Visual™ version 2.15 (Sophia Genetics, Massachusetts, USA), Sorting Intolerant form Tolerant (SIFT), MutationTaster, and PolyPhen-2, the population database gnomAD, and the variant databases HGMD® Professional 2020.3 (QIAGEN A/S, Aarhus, Denmark) and ClinVar. If the variant was located in an obligatory splice-site or presumed to lead to protein truncation, it was considered likely deleterious. For missense variants, we generated CLUSTAL alignments displaying the evolutionary conservation among orthologues in vertebrates and non-vertebrates using Clustal Omega and Ensembl Genome Browser (release 102). Missense variants were further assessed for deleteriousness using the prediction algorithms PolyPhen-2, SIFT, and MutationTaster. All variants were confirmed using Sanger sequencing. If parental DNA was available (especially for compound heterozygous or heterozygous variants to confirm segregation), parental DNA was also used for Sanger confirmation. Compound-heterozygous variants were only considered if parental DNA was available to us for segregation analysis and confirmation of a biallelic variant. The final decision on the deleteriousness of a newly identified variant was performed by a six-member panel of experienced nephro-geneticists.

**Web Resources**

1000 Genomes Browser, http://browser.1000genomes.org

ClinVar, https://www.ncbi.nlm.nih.gov/clinvar

Clustal Omega, http://www.ebi.ac.uk/Tools/msa/clustal

dbSNP, https://www.ncbi.nlm.nih.gov/snp

Ensembl Genome Browser, http://www.ensembl.org

Exome Variant Server, http://evs.gs.washington.edu/EVS

Genome Aggregation Database (gnomAD), http://gnomad.broadinstitute.org

HGMD® Professional 2020.4, https://portal.biobase-international.com/hgmd

Human Gene Nomenclature Committee, http://www.genenames.org/;

Mouse Genome Informatics, http://www.informatics.jax.org

MutationTaster, http://www.mutationtaster.org

Online Mendelian Inheritance in Man (OMIM®), http://www.omim.org

PolyPhen2, http://genetics.bwh.harvard.edu/pph2

Primer3, http://primer3.ut.ee/;

Sorting Intolerant From Tolerant (SIFT), http://sift.jcvi.org

UCSC Genome Browser, http://genome.ucsc.edu/cgi-bin/hgGateway

Uniprot Consortium, http://www.uniprot.org

VarSome, http://www.varsome.comPolyphen2, http://genetics.bwh.harvard.edu/pph2/;

Biobase, https://portal.biobase-international.com/hgmd/pro/search_gene.php?;

**Software**

CLC Genomics Workbench (6.5.1)™ (CLC-bio, Aarhus, Denmark)

Alamut Visual (2.7 rev. 1)® (Interactive Biosoftware, Rouen, France)
