# Supplementary material for: Exome sequencing identifies a likely causative variant in 53% of families with ciliopathy-related features on renal ultrasound after excluding NPHP1 deletions
Source: Genes Dis. 2023 Sep 15;11(5):101111. doi: 10.1016/j.gendis.2023.101111 (PMC11167256; doi:10.1016/j.gendis.2023.101111)
Supplement: Multimedia component 2 [file mmc2.docx]

**Table S1: 96 genes that represent monogenic causes of human cystic kidney disease or nephronophthisis-related ciliopathies if mutated, and that were evaluated in ES data of this study.**

| **Gene symbol** | **Alias** | **Protein** | **Reference** | **Mode of Inheritance** | **MIM Number** |
| --- | --- | --- | --- | --- | --- |
| *AHI1* | *JBTS3* | Abelson Helper Integration Site 1 | Parisi *J Med Gen* 43(4):334 ,2005 | AR | 608894 |
| *ALMS1* | *ALMS* | Altrom Syndrome Protein 1 | Collin *Nat Genet* 31(1):74, 2002 | AR | 606844 |
| *ANKS6* | *NPHP16* | Ankyrin repeat and sterile alpha motif domain containing 6 | Hoff *Nat Genet* 45(8):951, 2013 | AR | 615370 |
| *ARL13B* | *JBTS8* | ADP-ribosylation factot-like 13B | Cantagrel *Am J Hum Genet* 83(2):170, 2008 | AR | 608922 |
| *ARL6* | *BBS3* | Bardet-Biedl Syndrome 3 | Khaddour *Hum Mutat* 28(5), 523, 2007 | AR | 608845 |
| *B9D1* | *MKS9* | B9 domain containing protein 1 | Romani *Orphanet J Rare Dis* 9:72, 2014 | AR | 614144 |
| *B9D2* | *MKS10* | B9 domain containing protein2 | Dowdle *Am J Hum Genet* 89(1): 94, 2011 | AR | 611951 |
| *BBIP1* | *BBS18* | Bardet-Biedl syndrome 18 | Scheidecker *J Med Genet* 51(2):132, 2014 | AR | 613605 |
| *BBS1* | *BBS1* | Bardet-Biedl Syndrome 1 | Mykytyn *Nat Genet* 31(4):435, 2002 | AR | 209901 |
| *BBS10* | *BBS10* | Bardet-Biedl Syndrome 10 | Stoetzel *Nat Genet* 38(5):521, 2006 | AR | 610148 |
| *BBS12* | *BBS12* | Bardet-Biedl Syndrome 12 | Stoetzel *Am J Hum Genet* 80(1):1, 2007 | AR | 610683 |
| *BBS2* | *BBS2* | Bardet-Biedl Syndrome 2 | Katsanis *Science* 293(5538):2256, 2001 | AR | 606151 |
| *BBS4* | *BBS4* | Bardet-Biedl Syndrome 4 | Mykytyn *Nat Genet* 8(2):188, 2001 | AR | 600374 |
| *BBS5* | *BBS5* | Bardet-Biedl Syndrome 5 | Tieder *Int J Pediatr Nephrol* 3(3):199, 1982 | AR | 603650 |
| *BBS7* | *BBS7* | Bardet-Biedl Syndrome 7 | Badano *Am J Hum Genet* 72(3), 650, 2003 | AR | 607590 |
| *C2CD3* | *OFD14* | Orofaciodigital syndrome 14 | Thauvin-Robinet *Nature Genet* 46(8):905, 2014. | AR | 615944 |
| *C5orf42* | *JBTS17* | Chromosome 5 open reading frame 42 | Srour *Am J Hum Genet* 90(4):693, 2012 | AR | 614571 |
| *CC2D2A* | *JBTS9* | Coiled-coil and C2 domains-containing protein 2A | Noor *Am J Hum Genet* 82(4):1011, 2008 | AR | 612013 |
| *CCDC28B* |  | Coiled-coil domain containing 28B | Lee *Nat Genet* 44(2):193, 2014 | AR | 610162 |
| *CCDC41* | *CEP83* | Homeobox B8 | Failler *Am J Hum Genet* 94(6):905, 2014 | AR | 615847 |
| *CEP104* | *JBTS25* | Centrosomal protein 41kDa | Korvatska *Am J Med Genet* *B Neuropsychiatr Genet* 156B(3):303, 2011 | AR | 616690 |
| *CEP120* | *SRTD13* | Centrosomal protein 120kDa | Shaheen *Hum Mol Genet* 24(5):1410, 2015 | AR | 613446 |
| *CEP164* | *NPHP15* | Centrosomal protein 164kDa | Chaki *Cell* 150(3):533, 2012 | AR | 614848 |
| *CEP290* | *NPHP6* | Centrosomal protein 290kDa | Sayer *Nat Genet* 38(6):674, 2006 | AR | 610142 |
| *CEP41* | *TSGA14* | Homeobox B9 | Lee *Nat Genet* 44(2):193, 2012 | AR | 610523 |
| *CSPP1* | *JBTS21* | Centrosome spindle pole-associated protein 1 | Akizu *Am J Hum Genet* 94(1):80, 2014 | AR | 611654 |
| *DCDC2* | *NPHP19* | Double-cortin domain containing protein 2 | Schueler *Am J Hum Genet* 96(1):81, 2015 | AR | 605755 |
| *DDX59* | *OFD5* | Orofaciodigital syndrome 5 | Shamseldin *Am J Hum Genet* 93(3):555, 2013 | AR | 615464 |
| *DYNC2H1* | *SRTD3* | Dynein cystoplasmic 2 heavy chain | El Hokayem *J Med Genet* 49(4):227, 2012 | AR | 603297 |
| *EVC* |  | EvC Ciliary Complex Subunit 1 | Ruiz-Perez *Nat Genet* 24(3):283, 2000 | AR/AD | 604831 |
| *EVC2* |  | EvC Ciliary Complex Subunit 2 | Kurian *Indian* *J Dent Res* 18(1):31, 2007 | AR/AD | 607261 |
| *FAN1* | *MTMR15* | FANCI-associated nuclease 1 | Zhou *Nat Genet* 44(8):910, 2012 | AR | 613534 |
| *GLIS2* | *NPHP7* | GLIS Family Zinc Finger 2 | Attanasio *Nat Genet* 39(8):1018, 2007 | AR | 608539 |
| *HOXA4* |  | Hoemobox A4 | Acampora *Nucleic Acids Res* 17(24):10385, 1985 | AD | 142953 |
| *HOXB6* |  | Homeobox B6 | Kaur *J Exp Zool* 264(3):323, 1992 | AD | 142961 |
| *HSD17B4* | *MFP2* | Hydroxysteroid 17-beta dehydrogenase 4 | Lee *Nat Genet* 44(2):193, 2013 | AR | 601860 |
| *IFT122* | *CED1* | Intraflagellar transport 122 | Walczak-Sztulpa *Am J Hum Genet* 86(6):949, 2010 | AR | 606045 |
| *IFT140* | *SRTD9* | intraflagellar transport 140 | Perrault *Am J Hum Genet* 90(5):864, 2012 | AR | 614620 |
| *IFT172* | *NPHP17* | Intraflagellar transport 172 homologue (Chlamydomonas) | Halbritter *Am J Hum Genet* 93(5):915, 2013 | AR | 607386 |
| *IFT27* | *BBS20* | Bardet-Biedl Syndrome 20 | Schaefer *J Med Genet* 61(5):447 2016 | AR | 615870 |
| *IFT43* | *CED3* | intraflagellar transport 43 | Gilissen *Am J Hum Genet* 87(3):418, 2010 | AR | 614068 |
| *IFT52* | *SRTD* | Intraflagellar Transport 52 | Girisha *Clin Genet* 90(6):536, 2016 | AR | 617094 |
| *IFT57* |  | Intraflagellar Transport 57 | Bruel *J Med Genet* 54(6):371, 2017 | AR | 606621 |
| *IFT80* | *SRTD2* | Intraflagellar Transport 80 | Beales *Nat Genet* 39(6):727 2007 | AR | 611177 |
| *IFT81* | *CDV-1* | Intraflagellar Transport 81 | Perrault *J Med Genet* 52(10):657, 2015 | AR | 605489 |
| *INPP5E* | *JBTS1* | Inositol polyphosphate-5-phosphatase | Bielas *Nat Genet* 41(9):1032, 2009 | AR | 613037 |
| *INVS* | *NPHP2* | Inversin | Otto *Nat Genet* 34(4):413, 2003 | AR | 243305 |
| *IQCB1* | *NPHP5* | IQ motif containing B1 | Otto *Nat Genet* 37(3):282, 2005 | AR | 609237 |
| *KIAA0556* | *JBTS26* | Katanin-intereacting protein | Saunders *Genome Biol* 16:293, 2015 | AR | 616650 |
| *KIAA0586* | *JBTS23* | TALPID 3, chicken homolog of | Bachmann-Gagescu *Hum Mutat* 36(9):831, 2015 | AR | 610178 |
| *KIAA0753* | *OFD15* | Orofaciodigital syndrome 15 | Chevrier *Hum Mol Genet* 25(3):497, 2016 | AR | 617112 |
| *KIF14* | *MKS12* | Kinesin family member 14 | Filges *Clin Genet* 86(3):220, 2013 | AR | 611279 |
| *KIF7* | *JBTS12* | Kinesin family member 7 | Putoux *Nat Genet* 43(6):601, 2011 | AR | 611254 |
| *LZTFL1* | *BBS17* | Bardet-Biedl syndrome 17 | Marion *J Med Genet* 49(5):317, 2012 | AR | 606568 |
| *MAPKBP1* | *NPHP20* | Mitogen activated protein kinase-binding protein 1 | Macia *Am J Hum Genet* 100(2):323, 2017 | AR | 616786 |
| *MKKS* | *BBS6* | Bardet-Biedl Syndrome 6 | Katsanis *Nat Genet* 26(1):67, 2000 | AR | 604896 |
| *MKS1* | *MKS1* | Meckel syndrome, type 1 | Kyttälä *Nat Genet* 38(2):155, 2006 | AR | 609883 |
| *NEK1* | *SRTD6* | NIMA Related Kinase 1 | Thiel *Am J Hum Genet* 88(1):106, 2011 | AR | 604588 |
| *NEK8* | *NPHP9* | NIMA (never in mitosis gene a) - related kinase 8 | Otto *J Am Soc Nephrol* 19(3):587, 2008 | AR | 609799 |
| *NPHP1* | *NPHP1* | Nephrocystin 1 | Hildebrandt *Nat Genet* 17(2):149, 1997 | AR | 607100 |
| *NPHP3* |  | Nephrocystin 3 | Olbrich *Nat Genet* 34(4):455, 2003 | AR | 608002 |
| *NPHP4* |  | Nephronophthisis 4 | Otto *Am J Hum Genet* 71(5):1161, 2002 | AR | 607215 |
| *OFD1* |  | Orofaciodigital syndrome I | Feather *Hum Mol Genet* 6(7):1163, 1997 | XLD | 300170 |
| *PDE6D* | *JBTS22* | Phosphodiesterase 6D | Thomas *Hum Mutat* 35(1):137, 2014 | AR | 602676 |
| *PIK3R4* | *VPS15* | Phosphatidyllinositol 3-kinase, regulatorry subunit 4 | Panaretou *J Biol Chem* 272(4): 2477, 1997 | AR | 602610 |
| *PKHD1* | *ARPKD* | PKHD1, Fibrocystin/Polyductin | Bergmann *Kidney Int* 67(3):829 2005 | AR | 606702 |
| *POC1B* |  | Cone rod dystrophy 20 | Roosing *Am J Hum Genet* 95(2):131, 2014 | AR | 614784 |
| *PTHB1* | *BBS9* | Bardet-Biedl Syndrome 9 | Nishimura  *Am J Hum Genet* 77(6):1021, 2005 | AR | 607968 |
| *RPGRIP1L* | *NPHP8* | RPGRIP1 Like | Arts *Nat Genet* 39(7):882, 2007 | AR | 610937 |
| *SCLT1* | *OFD9* | Orofaciodigital syndrome 9 | Adly *Hum Mutation* 35(1): 36, 2013 | AR | 611399 |
| *SDCCAG8* | *NPHP10* | Serologically Defined Colon Cancer Antigen 8 | Otto *Nat Genet* 42(10):840, 2010 | AR | 613524 |
| *SLC41A1* |  | Solute carrier member family 41, member 1 | Hurd *J Am Soc Nephrol* 24(6):967, 2013 | AR | 610801 |
| *TBC1D32* |  | Homeobox B11 | Adly *Hum Mutat* 35:36, 2014 | AD | 615867 |
| *TCTN1* | *JBTS13* | tectonic family member 1 | Garcia-Gonzalo *Nat Genet* 43(8):776, 2011 | AR | 609863 |
| *TCTN2* | *JBTS24* | Tectonic family member 2 | Huppke *Eur J Hum Genet* 23(5):616, 2015 | AR | 613846 |
| *TCTN3* | *JBTS18* | Tectonic family member 3 | Thomas *Am J Hum Genet* 91(2):372, 2012 | AR | 613847 |
| *TMEM107* | *MKS13* | Transmembrane Protein 107 | Shasheen *Hum Mol Genet* 24(18):5211, 2015 | AR | 616183 |
| *TMEM138* | *JBTS16* | Transmembrane protein 138 | Lee *Science* 335(6071): 966, 2012 | AR | 614459 |
| *TMEM216* | *JBTS2* | Transmembrane Protein 216 | Edvardson *Am J Hum Genet* 86(1):93, 2010 | AR | 613277 |
| *TMEM231* | *JBTS20* | Transmembrane protein 231 | Srour *J Med Genet* 49: 636-641, 2012 | AR | 614949 |
| *TMEM237* | *JBTS14* | Transmembrane protein 237 | Huang *Am J Hum Genet* 89(6):713, 2011 | AR | 614423 |
| *TMEM67* | *NPHP11* | Transmembrane Protein 67 | Otto *J Med Genet* 46(10):663, 2009 | AR | 609884 |
| *TRAF3IP1* | *SLS9* | TNF receptor-associated factor 3-interacting protein 1 | Berbari *Dev Biol* 360(1):66, 2011 | AR | 607380 |
| *TRIM32* | *BBS11* | Bardet-Biedl Syndrome 11 | Chiang *Proc Natl Acad Sci* 103(16):6287,2006 | AR | 602290 |
| *TTC21B* | *NPHP12* | Tetratricopeptide Repeat Domain 21B | Davis *Nat Genet* 43(3):189, 2011 | AR | 612014 |
| *TTC8* | *BBS8* | Bardet-Biedl Syndrome 8 | Stoetzel *J Hum Genet* 51(1):81, 2005 | AR | 608132 |
| *TXNDC15* |  | Thioredoxin domain-containing protein 15 | Honjo *Cell Rep* 16(2):295, 2016 | AR | 617778 |
| *UMOD* |  | Uromodulin | Hart *J Med Genet* 39(12):882, 2002 | AD | 191845 |
| *USH2A* |  | Homeobox B10 | Smith *Genomics* 14(4):995, 1992 | AR | 608400 |
| *WDPCP* | *BBS15* | WD repeat-containing planar cell polarity effector | Stone *Nat Genet* 25(1):79, 2000 | AR | 613580 |
| *WDR19* | *NPHP13* | WD repeat domain 19 | Bedrup *Am J Hum Genet* 89(5):634, 2011 | AR | 608151 |
| *WDR34* | *SRTD11* | WD Repeat Domain 34 | Schmidts *Am J Hum Genet* 93(5):932, 2013 | AR | 613363 |
| *WDR35* | *CED2* | WD repeat domain 35 | Gilissen *Am J Hum Genet* 87(3):418, 2010 | AR | 613602 |
| *WDR60* | *SRTD8* | WD Repeat Domain 60 | McInerney-Leo *Am J Hum Genet* 93(3): 515, 2013 | AR | 615462 |
| *XPNPEP3* | *NPHPL1* | X-prolyl aminopeptidase 3 | O'Toole *J Clin Invest* 120(3):791, 2010 | AR | 613553 |
| *ZNF423* | *NPHP14* | Zinc finger protein 423 | Chaki *Cell* 150(3):533, 2012 | AR | 604557 |
