# Supplementary material for: Exome sequencing identifies a likely causative variant in 53% of families with ciliopathy-related features on renal ultrasound after excluding NPHP1 deletions
Source: Genes Dis. 2023 Sep 15;11(5):101111. doi: 10.1016/j.gendis.2023.101111 (PMC11167256; doi:10.1016/j.gendis.2023.101111)
Supplement: Multimedia component 3 [file mmc3.docx]

**Table S2. 84 genes which can mimic an NPHP-RC phenotype if mutated.**

| **Gene symbol** | **Protein** | **Reference** | **Mode of Inheritance** | **Disease Phenotype** | **MIM Number** |
| --- | --- | --- | --- | --- | --- |
| *ACE* | Angiotensin I-converting enzyme | Gribouval *Nat Genet* 37:964, 2005 | AR | Isolated CAKUT | 106180 |
| *AGT* | Angiotensinogen | Gribouval *Nat Genet* 37:964, 2005 | AR | Isolated CAKUT | 106150 |
| *AGTR1* | Angiotensin II receptor, type 1 | Gribouval *Nat Genet* 37:964, 2005 | AR | Isolated CAKUT | 106165 |
| *DSTYK* | Dual serine/threonine and tyrosine protein kinase | Sanna-Cherchi *N Engl J Med* 369:621, 2013 | AD | Isolated CAKUT | 612666 |
| *ETV4* | ETS translocation variant 4, E1A enhancer binding protein | Chen *IJPCH* 4:61, 2016 | AR | Isolated CAKUT | 600711 |
| *EYA1* | Eyes absent homolog 1 | Abdelhak *Nat Genet* 15:157, 1997 | AD | Isolated CAKUT | 601653 |
| *FGF20* | Fibroblast Growth Factor 20 | Barak *Dev Cell* 22:1191, 2012 | AR | Isolated CAKUT | 605558 |
| *FRAS1* | Extracellular matrix protein FRAS1 | Kohl *J Am Soc Nephrol* 25:1917, 2014 | AR | Isolated CAKUT | 607830 |
| *FREM1* | FRAS1 related extracellular matrix protein 1 | Kohl *J Am Soc Nephrol* 25:1917, 2014 | AR | Isolated CAKUT | 608944 |
| *FREM2* | FRAS1 related extracellular matrix protein 2 | Kohl *J Am Soc Nephrol* 25:1917, 2014 | AR | Isolated CAKUT | 608945 |
| *GATA3* | GATA binding protein 3 | Pandolfi *Nat Genet* 11:40, 1995 | AD | Isolated CAKUT | 131320 |
| *GRIP1* | Glutamate receptor interacting protein 1 | Kohl *J Am Soc Nephrol* 25:1917, 2014 | AR | Isolated CAKUT | 604597 |
| *HPSE2* | Heparanase 2 (Inactive) | Bulum *Nephron* 130:54, 2015 | AR | Isolated CAKUT | 613469 |
| *ITGA8* | Integrin α8 | Humbert *Am J Hum Genet* 189:1260, 2014 | AR | Isolated CAKUT | 604063 |
| *KAL1* | Anosmin 1 | Hardelin *Proc Natl Acad Sci* 89:8190, 1992 | XLR | Isolated CAKUT | 300836 |
| *MUC1* | Mucin 1 | Kirby *Nat Genet* 45:299, 2013 | AD | Isolated CAKUT | 158340 |
| *NRIP1* | Nuclear Receptor Interacting Protein 1 | Vivante *J Am Soc Nephrol* 28:2364, 2107 | AD | Isolated CAKUT | 602490 |
| *REN* | Renin | Gribouval *Nat Genet* 37:964, 2005 | AR | Isolated CAKUT | 179820 |
| *SALL1* | Sal-like protein 1 (also known as spalt-like transcription factor 1) | Kohlhase *Nat Gene*t 18:81, 1998 | AD | Isolated CAKUT | 602218 |
| *SIX1* | SIX homeobox 1 | Ruf *Proc Natl Acad Sci* 101: 8090, 2004 | AD | Isolated CAKUT | 601205 |
| *SIX2* | SIX homeobox 2 | Weber *J Am Soc Nephrol* 19:891, 2008 | AD | Isolated CAKUT | 604994 |
| *SOX17* | Transcription factor SIX-17 | Gimelli *Hum Mut* 31:1352, 2010 | AD | Isolated CAKUT | 610928 |
| *TBX18* | T-Box transcription factor | Vivante *Am J Hum Genet* 97:291, 2015 | AD | Isolated CAKUT | 604613 |
| *WNT4* | Protein Wnt-4 | Biason-Lauber *N Engl J Med* 351:792, 2004 | AD | Isolated CAKUT | 603490 |
| *ATXN10* | Ataxin 10 | Matsuura *Nat Genet* 26:191, 2000 | AD | Syndromic CAKUT | 611150 |
| *BMP7* | Bone Morphogenetic Protein 7 | Hwang *Kidney Int* 85:1429, 2014 | AD | Syndromic CAKUT | 112267 |
| *HNF1B* | Hepatocyte Nuclear Factor 1 beta | Bellanné-Chantelot *Ann Intern Med* 140:510, 2004 | AD | Syndromic CAKUT | 189907 |
| *LRIG2* | Leucione rich repeats and immunoglobulin like  domains containing protein 2 | Stuart *Am J Hum Genet* 92:259, 2013 | AR | Syndromic CAKUT | 608869 |
| *PAX2* | Paired box protein Pax-2 | Amiel *Eur J Hum Genet* 8:820, 2000 | AD | Syndromic CAKUT | 167409 |
| *POC1A* | POC1 centriolar protein | Shaheen *Am J Hum Genet* 91:330, 2012 | AR | Syndromic CAKUT | 614783 |
| *RERE* | Arginine-glutamic acid dipeptide repeats protein | Fregeau *Am J Hum Genet* 98:963, 2016 | AD | Syndromic CAKUT | 605226 |
| *SIX5* | Homeobox protein SIX5 | Hoskins *Am J Hum Genet* 80:800, 2007 | AD | Syndromic CAKUT | 600963 |
| *CRB2* | Crumbs, Drosphilia, Homolog of 2 | Ebarasi *Am J Hum Genet* 96: 153-161, 2015 | AR | Nephrotic Syndrome | 609720 |
| *COL4A3* | Collagen type IV alpha 3 chain | Lemmink *Hum Mol Genet* 3:1269, 1994 | AR/AD | Alport-Syndrome | 120070 |
| *COL4A4* | Collagen type IV alpha 4 chain | Mochizuki *Nat Genet* 8:77, 1994 | AR/AD | Alport-Syndrome | 120131 |
| *COL4A5* | Collagen type IV alpha 5 chain | Antignac *J Clin Invest* 93:1195, 1994 | XLD | Alport-Syndrome | 303630 |
| *AGXT* | Alanine-glyoxylate aminotransferase | Purdue *Proc Natl Acad Sci* 88:10900, 1991 | AR | NC/NL | 604285 |
| *ATP6V0A4* | ATPase, H+ transporting, lysosomal V0 subunit a4 | Smith *Nat Genet* 26:71, 2000 | AR | NC/NL | 605239 |
| *ATP6V1B1* | ATPase, H+ transporting, lysosomal 56/58kDa, V1 subunit B1 | Karet *Nat Genet* 21:84, 1999 | AR | NC/NL | 192132 |
| *CASR* | Calcium-sensing receptor | Pearce *N Engl J Med* 335:1115, 1996 | AD/AR | NC/NL | 601199 |
| *CLCN5* | Chloride channel, voltage-sensitive 5 | Lloyd *Nature* 379:445, 1996 | XLR | NC/NL | 300008 |
| *CLCNKB* | Chloride channel, voltage-sensitive Kb | Simon *Nat Genet* 17:171, 1997 | AR | NC/NL | 602023 |
| *CLDN16* | Claudin 16 | Simon *Science* 285:103, 1999 | AR | NC/NL | 603959 |
| *CLDN19* | Claudin 19 | Konrad *Am J Hum Genet* 79:949, 2006 | AR | NC/NL | 610036 |
| *CTNS* | Cystinosin | Town *Nat Genet* 18:319, 1998 | AR | NC/NL | 606272 |
| *CYP24A1* | Cytochrome P450, family 24, subfamily A, polypeptide 1 | Schlingmann *N Engl J Med* 36:410, 2011 | AR | NC/NL | 126065 |
| *GRHPR* | Glyoxylate reductase/hydroxypyruvate | Cramer *Hum Mol Genet* 8:2063, 1999 | AR | NC/NL | 604296 |
| *HNF4A* | Hepatocyte nuclear factor 4, alpha | Hamilton *J Med Genet* 51:165, 2014 | AD | NC/NL | 600281 |
| *HOGA1* | 4-hydroxy-2-oxoglutarate aldolase 1 | Belostotsky *Am J Hum Genet* 87:392, 2010 | AR | NC/NL | 613597 |
| *KCNJ1* | Potassium inwardly-rectifying channel, subfamily J, member 1 | Simon *Nat Genet* 14:152, 1996 | AR | NC/NL | 600359 |
| *KCNJ10* | Potassium Channel inwardly rectifyinh subfamily J, member 10 | Bockenhauer *N Engl J Med* 360:1960, 2009 | AR | NC/NL | 602208 |
| *OCRL* | Oculocerebrorenal syndrome of Lowe | Reilly *Am J Hum Genet* 42:748, 1988 | XLR | NC/NL | 300535 |
| *SLC12A1* | Solute carrier family 12, member 1 | Simon *Nat Genet* 13:183, 1996 | AR | NC/NL | 600839 |
| *SLC26A1* | Solute carrier family 26 (sulfate transporter), member 1 | Gee *Am J Hum Genet* 98:1228, 2016 | AR | NC/NL | 610130 |
| *SLC26A6* | Solute carrier family 26 member 6 | Jiang *Nat Genet* 38:474, 2006 | AR | NC/NL | 610068 |
| *SLC34A1* | Solute carrier family 34 | Prie *N Engl J Med* 347:983, 2002 | AD/AR | NC/NL | 182309 |
| *SLC34A3* | Solute carrier family 34 (sodium | Lorenz-Depiereux *Am J Hum Genet* 78:193, 2006 | AR | NC/NL | 609826 |
| *SLC3A1* | Solute carrier family 3, member 1  (cystine, dibasic and neutral amino acid transporters, activator of cystine, dibasic and neutral amino acid transport | Calonge *Nat Genet* 6:420, 1994 | AD/AR | NC/NL | 104614 |
| *SLC4A1* | Solute carrier family 4, anion exchanger, member 1  (erythrocyte membrane) | Bruce *J Clin Invest* 100:1693, 1997 | AD/AR | NC/NL | 109270 |
| *SLC7A9* | Solute carrier family 7 (glycoprotein associated | Feliubadalo *Nat Genet* 23:52, 1999 | AD/AR | NC/NL | 604144 |
| *SLC9A3R1* | Solute carrier family 9, subfamily A  (NHE3, cation proton antiporter 3), member 3 regulator 1 | Karim *N Engl J Med* 359:1128, 2008 | AD | NC/NL | 604990 |
| *VDR* | Vitamin D (1,25- dihydroxyvitamin D3) receptor | Scott *J Am Soc Nephrol* 10:1007, 1999 | AD/AR | NC/NL | 601769 |
| *XDH* | Xanthine dehydrogenase | Ichida *J Clin Invest* 99:2391, 1997. | AR | NC/NL | 607633 |
| *AQP2* | Aquaporin 2 | Knoers Europ *J Pediat* 150:370, 1991 | AR/AD | Tubulopathies | 107777 |
| *AVP* | Arginine Vasopressin | Abbes *Clin Chem* 46:1699, 2000 | AD | Tubulopathies | 192340 |
| *AVPR2* | Arginine Vasopressin Receptor 2 | Feldman *N Engl J Med* 352: 1884-1890, 2005 | XLR | Tubulopathies | 300538 |
| *BSND* | Barttin CLCNK Type Accessory Beta Subunit | Birkenhager *Nat Genet* 29:310, 2001 | AR | Tubulopathies | 606412 |
| *CYP27B1* | Cytochrome P450 Family 27 Subfamily B Member 1 | Wang *Am J Hum Genet* 63:1694, 1998 | AR | Tubulopathies | 609506 |
| *EGF* | Epidermal Growth Factor | Groenestege *J Clin Invest* 117:2260, 2007 | AR | Tubulopathies | 131530 |
| *FXYD2* | FXYD domain containing ion transport regulator 2 | de Baij *Nephrol Dial Transplant* 30:952, 2015 | AD | Tubulopathies | 601814 |
| *GNA11* | G Protein Subunit Alpha 11 | Li *J Clin Endocr Metab* 99:E1774, 2014 | AD | Tubulopathies | 139313 |
| *MMAA* | Methylmalonic aciduria type A protein | Dobson *Proc Nat Acad Sci* 99:15554, 2002 | AR | Tubulopathies | 607481 |
| *NR3C2* | Nuclear Receptor Subfamily 3 Group C Member 2 | Riepe *J Clin Endocr Metab* 91:4552, 2006 | AD | Tubulopathies | 600983 |
| *SCNN1A* | Sodium Channel Epithelial 1 Alpha Subunit | Chang *Nat Genet* 12:248, 1996 | AR | Tubulopathies | 600228 |
| *SCNN1B* | Sodium Channel Epithelial 1 Beta Subunit | Chang *Nat Genet* 12:248, 1996 | AR | Tubulopathies | 600760 |
| *SCNN1G* | Sodium Channel Epithelial 1 Gamma Subunit | Hansson *Nat Genet* 11:76, 1995 | AR/AD | Tubulopathies | 600761 |
| *SLC12A3* | Solute Carrier Family 12 Member 3 | Ng *Neurology* 67:1080, 2006 | AR | Tubulopathies | 600968 |
| *SLC2A2* | Solute Carrier Family 2 Member 2 | Manz *Pediatr Nephrol* 1:509, 1987 | AR | Tubulopathies | 138160 |
| *SLC4A4* | Solute Carrier Family 4 Member 4 | Igarashi *J Am Soc Nephrol* 12:713, 2001 | AR | Tubulopathies | 603345 |
| *SLC6A19* | Solute Carrier Family 6 Member 19 | Kleta *Nat Genet* 36:999, 2004 | AR | Tubulopathies | 608893 |
| *SLC7A7* | Solute Carrier Family 7 Member 7 | Borsani *Nature Genet* 21:297, 1999 | AR | Tubulopathies | 603593 |
| *TRPM6* | Transient Receptor Potential Cation Channel Subfamily M Member 6 | Schlingmann *Nature Genet* 31:166, 2002 | AR | Tubulopathies | 607009 |
| *WNK1* | WNK Lysine Deficient Protein Kinase 1 | Lafreniere *Am J Hum Genet* 74:1064, 2004 | AR | Tubulopathies | 605232 |
| *WNK4* | WNK Lysine Deficient Protein Kinase 4 | Wilson *Science* 293:1107, 2001 | AR | Tubulopathies | 601844 |

**Table S2. 84 genes which can mimic an NPHP-RC phenotype if mutated.** **AD** autosomal-dominant; **AR** autosomal-recessive; **CAKUT** congenital anomalies of the kidney and urinary tract; **MIM** mendelian inheritance in men; **NC/NL** nephrocalcinosis/nephrolithiasis; **XLD** X-linked dominant; **XLR** X-linked recessive
