# Supplementary material for: Exome sequencing identifies a likely causative variant in 53% of families with ciliopathy-related features on renal ultrasound after excluding NPHP1 deletions
Source: Genes Dis. 2023 Sep 15;11(5):101111. doi: 10.1016/j.gendis.2023.101111 (PMC11167256; doi:10.1016/j.gendis.2023.101111)
Supplement: Multimedia component 4 [file mmc4.docx]

## Table S3: Detailed information on phenotype and genotype for 45 families with likely causative variants in genes known to cause nephronophthisis-related ciliopathies (NPHP-RC).

| **Family** | **Gene** | **Zygo-**  **sity** | **Exon** | **Accession No Nt Change AA Change dbSNP** | **AA**  **Conser- vation** | **SIFT MT PP2** | **gnomAD** | **ClinVar HGMD ACMG** | **Refe-rence** | **Renal  Pheno-**  **type** | **Extra-**  **renal  Pheno-type** | **Ethnicity** | **Sex** | **Mbp  Homo-zygosity** |
| --- | --- | --- | --- | --- | --- | --- | --- | --- | --- | --- | --- | --- | --- | --- |
| **F1310** | ***SDCCAG8*** | **het** | **3** | **NM_001350248.1 c.278C>T p.(Pro93Leu) rs140413256** | **Ci** | **Del n/a 0.944** | **1/279/**  **282692** | **VUS - VUS** | **Novel** | **IE, C** | **-** | **Turkish** | **m** | **172.9** |
|  | ***SDCCAG8*** | **het** | **8** | **NM_001350248.1 c.791A>C p.(Tyr264Ser) rs747968552** | **Ci** | **Del n/a 0.331** | **0/5/**  **250896** | **- DM at c.784 VUS** | **^1^** |  |  |  |  |  |
| **A4977** | ***TMEM231*** | **hom** | **2** | **NM_001077416.2 c.532C>G p.(Pro178Ala) rs1442638461** | **Ci** | **Tol Dis 0.997** | **0/11/**  **169644** | **P DM VUS** | **^2^** | **IE** | **RP, ID, SS, CVA** | **Cauca-sian** | **f** | **30.9** |
| **A5109** | ***BBS2*** | **hom** | **5** | **NM_031885.3 c.565C>T p.(Arg189*) rs1273181642** | **Ns** | **n/a n/a n/a** | **0/2/**  **251318** | **P DM P** | **^3^** | **IE, C** | **MC, ID, FD, PD** | **Arabic** | **m** | **223.7** |
| **A5172** | ***IFT27*** | **hom** | **4** | **NM_001177701.2 c.200G>T p.(Gly67Val) n/a** | **Ci** | **Del Dis 1** | **n/a** | **- - VUS** | **Novel** | **IE** | **RP, ID, PD** | **Arabic** | **f** | **209.8** |
| **B1470** | ***PKHD1*** | **het** | **37** | **NM_138694.3 c.5912G>A p.(Gly1971Asp) rs180675584** | **Dr** | **Del Dis 1** | **0/11/**  **282408** | **P DM P** | **^4^** | **C** | **HF** | **Cauca-sian** | **m** | **11.3** |
|  | ***PKHD1*** | **het** | **27** | **NM_138694.3 c.2864T>G p.(Phe955Cys) rs777158800** | **Dr** | **Del Dis 1** | **0/4/**  **250766** | **- DM at c.2854 LP** | **^5^** |  |  |  |  |  |
| **B1601** | ***NPHP3*** | **het** | **21** | **NM_153240.4 c.2960C>T p.(Ser987Phe) n/a** | **Dr** | **Del Dis 0.994** | **n/a** | **- - VUS** | **Novel** | **IE** | **HF** | **Indian** | **f** | **8.4** |
|  | ***NPHP3*** | **het** | **19** | **NM_153240.4 c.2670_2671insT p.(Val891fs*7) n/a** | **Fs** | **n/a n/a n/a** | **n/a** | **- - P** | **Novel** |  |  |  |  |  |
| **B1620** | ***AHI1*** | **hom** | **16** | **NM_001134831.1 c.2212C>T p.Arg738* rs372659908** | **Ns** | **n/a n/a n/a** | **0/4/**  **280348** | **P DM P** | **^6^** | **C** | **RP, SS, GDD** | **Arabic** | **f** | **205.5** |
| **B1696** | ***NPHP3*** | **het** | **22** | **NM_153240.4 c.3157dup p.(Ser1053Phefs*3) n/a** | **Fs** | **n/a n/a n/a** | **n/a** | **- - P** | **Novel** | **C** | **-** | **Vietna-mese** | **m** | **5.3** |
|  | ***NPHP3*** | **het** | **Intron 19** | **NM_153240.4 c.2694-2_2694-1del n/a rs751527253** | **Sp** | **n/a n/a n/a** | **0/78/**  **282714** | **P DM P** | **^7^** |  |  |  |  |  |
| **B1716** | ***PKHD1*** | **hom** | **32** | **NM_138694.3 c.3710G>A p.(Cys1237Tyr) n/a** | **Gg** | **Del Dis 0.999** | **n/a** | **- DM LP** | **^8^** | **C** | **-** | **Arabic** | **f** | **140.5** |
| **B1734** | ***BBS7*** | **het** | **18** | **NM_176824.2 c.1967T>C p.(Leu656Pro) rs758219185** | **Ce** | **Del Dis 1** | **0/25/**  **282736** | **- - VUS** | **Novel** | **IE, C** | **-** | **Cauca-sian** | **m** | **4.4** |
|  | ***BBS7*** | **het** | **7** | **NM_176824.2 c.280A>C p.(Thr94Pro) n/a** | **Ci** | **Del Dis 0.992** | **n/a** | **- - VUS** | **Novel** |  |  |  |  |  |
| **B1751** | ***PKHD1*** | **hom** | **23** | **NM_138694.3 c.2369G>A p.(Gly790Glu) n/a** | **Ci** | **Del Dis 0.99** | **n/a** | **- - VUS** | **Novel** | **C** | **-** | **Arabic** | **f** | **163.5** |
| **B1752** | ***PKHD1*** | **het** | **36** | **NM_138694.3 c.5869G>A p.(Asp1957Asn) rs200929620** | **Xt** | **Tol Dis 0.526** | **0/1/**  **251168** | **- DM LP** | **^9^** | **C** | **-** | **Arabic** | **f** | **91.6** |
|  | ***PKHD1*** | **het** | **26** | **NM_138694.3 c.2733del p.(Asn911Lysfs*36) n/a** | **Fs** | **n/a n/a n/a** | **n/a** | **- - LP** | **Novel** |  |  |  |  |  |
| **B1786**  **(AS)** | ***IQCB1*** | **hom** | **14** | **NM_001023570.2 c.1479C>A p.(Tyr493*) n/a** | **Ns** | **n/a n/a n/a** | **n/a** | **- DM P** | **^10^** | **none** | **LCA** | **Arabic** | **m** | **137.8** |
| **B1786 (IP)** | ***IQCB1*** | **hom** | **14** | **NM_001023570.2 c.1479C>A p.(Tyr493*) n/a** | **Ns** | **n/a n/a n/a** | **n/a** | **- DM P** | **^10^** | **IE** | **LCA** | **Arabic** | **m** | **116.7** |
| **B1868**  **(IP)** | ***PKHD1*** | **hom** | **32** | **NM_138694.3 c.4870C>T p.(Arg1624Trp) rs200391019** | **Hs** | **Del Poly 0.729** | **0/39/**  **282816** | **P DM LP** | **^11^** | **C** | **-** | **Arabic** | **f** | **111.0** |
| **B1868 (AS)** | ***PKHD1*** | **hom** | **32** | **NM_138694.3 c.4870C>T p.(Arg1624Trp) rs200391019** | **Hs** | **Del Poly 0.729** | **0/39/**  **282816** | **P DM LP** | **^11^** | **C, IE** | **HF** | **Arabic** | **f** | **249.0** |
| **B2385** | ***NPHP3*** | **hom** | **Intron 13** | **NM_153240.4 c.1985+5G>A n/a rs754508002** | **Sp** | **n/a n/a n/a** | **0/2/**  **250212** | **P DM VUS** | **^7^** | **C** | **-** | **Vietna-mese** | **f** | **28.5** |
| **B2496** | ***BBS12*** | **hom** | **3** | **NM_001178007.1 c.30_34del p.(Arg11Thrfs*30) n/a** | **Fs** | **n/a n/a n/a** | **n/a** | **- - P** | **Novel** | **IE** | **PD, RP, ID** | **Arabic** | **m** | **94.4** |
| **B2584** | ***INVS*** | **hom** | **14** | **NM_014425.4 c.2719C>T p.(Arg907*) rs267607185** | **Ns** | **n/a n/a n/a** | **0/4/**  **251354** | **P DM P** | **^12^** | **IE, LCD** | **SS** | **Arabic** | **m** | **244.9** |
| **B2610 (IP w/ B2614)** | ***CEP290*** | **hom** | **Intron 23** | **NM_025114.3 c.2483+1G>T n/a n/a** | **Sp** | **n/a n/a n/a** | **n/a** | **- - P** | **Novel** | **IE** | **SS** | **Pakistani** | **m** | **141.2** |
| **B2613** | ***NPHP4*** | **hom** | **Intron 21** | **NM_015102.3 c.3045-1G>T n/a rs751011207** | **Sp** | **n/a n/a n/a** | **0/1/**  **244414** | **- - P** | **Novel** | **C, IE, LCD** | **SS** | **Pakistani** | **m** | **151.0** |
| **B2614**  **(IP w/ B2610)** | ***CEP290*** | **hom** | **Intron 23** | **NM_025114.3 c.2483+1G>T n/a n/a** | **Sp** | **n/a n/a n/a** | **n/a** | **- - P** | **Novel** | **IE, LCD** | **SS** | **Pakistani** | **m** | **165.3** |
| **B2686** | ***CEP290*** | **hom** | **41** | **NM_025114.3 c.5668G>T p.(Gly1890*) rs137852832** | **Ns** | **n/a n/a n/a** | **0/23/**  **242464** | **P DM P** | **^13^** | **IE, LCD** | **JS** | **Arabic** | **f** | **104.7** |
| **B2688** | ***PKHD1*** | **hom** | **32** | **NM_138694.3 c.4870C>T p.(Arg1624Trp) rs200391019** | **Hs** | **Del Poly 0.729** | **0/40/**  **282816** | **P DM LP** | **^11^** | **C** | **-** | **Arabic** | **f** | **170.7** |
| **B2715** | ***PKD2*** | **het** | **14** | **NM_000297.3 c.2614C>T p.(Arg872*) rs755226061** | **Ns** | **n/a n/a n/a** | **0/1/**  **251096** | **P DM P** | **^14^** | **C** | **-** | **Cauca-sian** | **m** | **6.4** |
| **B2728 (IP w/ B3812)** | ***PKHD1*** | **hom** | **32** | **NM_138694.3 c.4870C>T p.(Arg1624Trp) rs200391019** | **Hs** | **Del Poly 0.729** | **0/39/**  **282816** | **P DM LP** | **^11^** | **IE, C** | **-** | **Arabic** | **f** | **178.0** |
| **B2757** | ***PKHD1*** | **hom** | **32** | **NM_138694.3 c.4870C>T p.(Arg1624Trp) rs200391019** | **Hs** | **Del Poly 0.729** | **0/39/**  **282816** | **P DM LP** | **^11^** | **IE, C** | **HF** | **Arabic** | **f** | **5.6** |
| **B2788** | ***PKHD1*** | **het** | **32** | **NM_138694.3 c.4993C>T p.(Gln1665*) n/a** | **Ns** | **n/a n/a n/a** | **n/a** | **- - P** | **Novel** | **IE, C** | **GR, HF** | **Arabic** | **m** | **91.5** |
|  | ***PKHD1*** | **het** | **32** | **NM_138694.3 c.4870C>T p.(Arg1624Trp) rs200391019** | **Hs** | **Del Poly 0.729** | **0/39/**  **282816** | **P DM LP** | **^11^** |  |  |  |  |  |
| **B2857** | ***PKHD1*** | **hom** | **6** | **NM_138694.3 c.5751G>A p.(Gln1917=) rs398124489** | **Sp** | **n/a n/a n/a** | **0/4/**  **282572** | **VUS DM at c.5750 VUS** | **^15^** | **C** | **FD, MC, ID, D,  RP, GR, SD, PD,  CHD** | **Pakistani** | **m** | **66** |
| **B2859** | ***IFT172*** | **het** | **46** | **NM_015662.2 c.4960A>G p.(Met1654Val) rs529850410** | **Dm** | **Tol n/a 0.834** | **0/44/**  **248000** | **- DM at c.4964 LB** | **^16^** | **C** | **-** | **Pakistani** | **m** | **4.3** |
|  | ***IFT172*** | **het** | **30** | **NM_015662.2 c.3268G>A p.(Val1090Met) rs76076247** | **Dm** | **Del n/a 0.997** | **0/50/**  **282746** | **VUS - LB** | **Novel** |  |  |  |  |  |
| **B3049** | ***PKHD1*** | **hom** | **32** | **NM_138694.3 c.4870C>T p.(Arg1624Trp) rs200391019** | **Hs** | **Del Poly 0.729** | **0/39/**  **282816** | **P DM LP** | **^11^** | **C** | **-** | **Indone-sian** | **m** | **78.2** |
| **B3073** | ***PKD1*** | **het** | **43** | **NM_001009944.2 c.11863C>T p.(Gln3955*) n/a** | **Ns** | **n/a n/a n/a** | **n/a** | **P DM P** | **^17^** | **C** | **-** | **Arabic** | **f** | **8.3** |
| **B3252** | ***NPHP1*** | **hom** | **2** | **NM_000272.3 c.143G>A p.(Arg48Lys) rs779393628** | **Dr** | **Tol Dis 0.987** | **0/1/**  **245518** | **- DM VUS** | **^1^** | **IE, LCD** | **SS** | **Arabic** | **f** | **6.6** |
| **B3261** | ***INPP5E*** | **hom** | **6** | **NM_019892.4 c.1304G>A p.(Arg435Gln) rs121918129** | **Dm** | **Del Dis 1** | **0/5/**  **156220** | **P DM LP** | **^18^** | **C** | **ID, RP, GR, SD** | **Arabic** | **m** | **12.7** |
| **B3262** | ***PKHD1*** | **het** | **Intron 7** | **NM_138694.3 c.528-2A>G n/a n/a** | **Sp** | **n/a n/a n/a** | **n/a** | **- DM P** | **^5^** | **C** | **-** | **Arabic** | **f** | **17.0** |
|  | ***PKHD1*** | **het** | **7** | **NM_138694.3 c.525del p.(Asp175Glufs*4) n/a** | **Fs** | **n/a n/a n/a** | **n/a** | **P - P** | **Novel** |  |  |  |  |  |
| **B3302** | ***PKHD1*** | **het** | **32** | **NM_138694.3 c.4492_4494delinsAG p.(Leu1499*) n/a** | **Ns** | **n/a n/a n/a** | **n/a** | **P - P** | **Novel** | **IE, C** | **PS, HF** | **Cauca-**  **sian** | **m** | **6.3** |
|  | ***PKHD1*** | **het** | **3** | **NM_138694.3 c.107C>T p.(Thr36Met) rs137852944** | **Dr** | **Del Dis 0.972** | **0/144/**  **282706** | **P DM P** | **^15^** |  |  |  |  |  |
| **B3406** | ***PKHD1*** | **het** | **57** | **NM_138694.3 c.8870T>C p.(Ile2957Thr) rs760222236** | **Ci** | **Del Dis 0.91** | **0/15/**  **282526** | **P DM P** | **^15^** | **IE, C** | **-** | **Cauca-sian** | **m** | **12.5** |
|  | ***PKHD1*** | **het** | **3** | **NM_138694.3 c.107C>T p.(Thr36Met) rs137852944** | **Dr** | **Del Dis 0.972** | **0/144/**  **282706** | **P DM P** | **^15^** |  |  |  |  |  |
| **B3547x** | ***BBS4*** | **hom** | **Intron 3** | **NM_033028.4 c.157-2A>G n/a rs113994192** | **Sp** | **n/a n/a n/a** | **0/1/**  **251008** | **P DM P** | **^19^** | **C, LCD** | **PD, LE** | **Arabic** | **f** | **82.7** |
| **B3636** | ***PKHD1*** | **hom** | **3** | **NM_138694.3 c.107C>T p.(Thr36Met) rs137852944** | **Dr** | **Del Dis 0.972** | **0/144/**  **282706** | **P DM P** | **^15^** | **C** | **GR, SD** | **Arabic** | **f** | **279.5** |
| **B3694** | ***NPHP4*** | **hom** | **15** | **NM_015102.3 c.1843C>T p.(Gln615*) rs372569805** | **Ns** | **n/a n/a n/a** | **0/1/**  **249232** | **- - P** | **Novel** | **IE, LCD** | **RP, SS** | **Pakistani** | **f** | **133.8** |
| **B3696** | ***CEP290*** | **hom** | **23** | **NM_025114.3 c.2451_2453del p.(His817del) n/a** | **Dr** | **n/a n/a n/a** | **n/a** | **- DM at c.2457 VUS** | **^1^** | **IE, C, LCD** | **RP, SS** | **Pakistani** | **m** | **320.0** |
| **B3754** | ***WDR19*** | **het** | **28** | **NM_025132.3 c.3184-2A>C n/a rs1020915921** | **Sp** | **n/a n/a n/a** | **n/a** | **P - P** | **Novel** | **IE** | **D, SS, N** | **Brazilian** | **m** | **0.1** |
|  | ***WDR19*** | **het** | **32** | **NM_025132.3 c.3533G>A p.(Arg1178Gln) rs79436363** | **Ce** | **Tol Dis 0.948** | **0/17/**  **267854** | **P DM P** | **^1^** |  |  |  |  |  |
| **B3811** | ***CEP290*** | **hom** | **41** | **NM_025114.3 c.5668G>T p.(Gly1890*) rs137852832** | **Ns** | **n/a n/a n/a** | **0/23/**  **242464** | **P DM P** | **^13^** | **IE** | **D, RP, ID** | **Arabic** | **m** | **161.1** |
| **B3812 (IP w/ B2728)** | ***PKHD1*** | **hom** | **32** | **NM_138694.3 c.4870C>T p.(Arg1624Trp) rs200391019** | **Hs** | **Del Poly 0.729** | **0/40/**  **282816** | **P DM LP** | **^11^** | **C** | **-** | **Arabic** | **m** | **231.5** |
| **B3868** | ***NPHP1*** | **hom** | **8** | **NM_000272.3 c.738_739del p.(His247Leufs*18) n/a** | **Fs** | **n/a n/a n/a** | **n/a** | **- - P** | **Novel** | **IE, C** | **-** | **Cauca-sian** | **f** | **6.5** |
| **B3909** | ***TMEM231*** | **het** | **2** | **NM_001077416.2 c.532C>G p.(Pro178Ala) rs1442638461** | **Ci** | **Tol Dis 0.997** | **0/11/**  **169644** | **P DM VUS** | **^2^** | **IE** | **HF, RP, ID** | **Cauca-sian** | **f** | **5.6** |
|  | ***TMEM231*** | **het** | **1** | **NM_001077416.2 c.400C>T p.(Leu134Phe) rs376555896** | **Ci** | **Tol Dis 0.992** | **0/4/**  **212802** | **LP DM LP** | **^2^** |  |  |  |  |  |
| **B3976** | ***MKKS*** | **hom** | **6** | **NM_018848.3 c.1436C>G p.(Ser479*) rs753180214** | **Ns** | **n/a n/a n/a** | **0/4/**  **251408** | **- DM P** | **^20^** | **IE, C** | **MS, PD** | **Cauca-sian** | **f** | **17.6** |
| **B4068** | ***ANKS6*** | **hom** | **Intron 3** | **NM_173551.3 c.907+2T>A n/a rs1438673595** | **Sp** | **n/a n/a n/a** | **0/1/**  **248226** | **P DM P** | **^21^** | **IE, LCD** | **HC, FD, MC, GR,  SD, S** | **Arabic** | **m** | **265.6** |
| **B4128** | ***NPHP4*** | **hom** | **Intron 14** | **NM_015102.3 c.1764-1G>T n/a n/a** | **Sp** | **n/a n/a n/a** | **n/a** | **- - P** | **Novel** | **IE, LCD** | **-** | **Arabic** | **m** | **154.8** |

**Table S3.** Detailed information on phenotype and genotype for 45 families with likely causative variants in genes known to cause nephronophthisis-related ciliopathies (NPHP-RC). **AA** amino acid; **ACMG** American College of Medical Genetics; **AS** affected sibling; **B** benign; **C** cysts; **Ce** *Caenorhabditis elegans*; **CHD** congenital heart defect; **Ci** *Ciona intestinalis*; **CVA** cerebellar vermis aplasia; **D** deafness; **Del** deleterious; **Dis** disease-causing; **DM** disease-causing mutation; **Dm** *Drosophila melanogaster*; **Dr** *Danio rerio*; **FD** facial dysmorphism; **Fs** Frameshift; **GDD** global developmental delay; **Gg** *Gallus gallus*; **GR** growth retardation; **HC** hepatic cysts; **het** heterozygous; **HF** hepatic fibrosis; **HGMD** Human Genome Mutation Database; **hom** homozygous; **Hs** *Homo sapiens*; **ID** intellectual disability; **IE** Increased echogenicity; **IP** Index Patient; **JS** Joubert Syndrome; **LB** likely benign; **LCA** leber congenital amaurosis; **LCD** loss of corticomedullary differentiation; **LE** low-set ears; **LP** likely pathogenic; **m** male; **Mbp** Megabasepairs; **MC** microcephaly; **MS** macrosomia; **MT** mutation taster; **N** nystagmus; **n/a** not available; **Ns** Nonsense; **Nt** nucleotide; **P** pathogenic; **PD** polydactyly; **Poly** polymorphism; **PP2** Polyphen2; **PS** pyloric stenosis; **RP** retinitis pigmentosa; **S** seizures; **SD** skeletal deformity; **SIFT** Sorting Intolerant From Tolerant; **Sp** Splice site; **SS** short stature; **Tol** tolerated; **VUS** variant of uncertain significange; **Xt** *Xenopus tropicalis*.

**References**

1. Halbritter J, Porath JD, Diaz KA, et al. Identification of 99 novel mutations in a worldwide cohort of 1,056 patients with a nephronophthisis related ciliopathy. *Hum Genet.* 2013;132(8):865-884.

2. Braun DA, Schueler M, Halbritter J, et al. Whole exome sequencing identifies causative mutations in the majority of consanguineous or familial cases with childhood-onset increased renal echogenicity. *Kidney Int.* 2016;89(2):468-475.

3. Smaoui N, Chaabouni M, Sergeev YV, et al. Screening of the eight BBS genes in Tunisian families: no evidence of triallelism. *Invest Ophthalmol Vis Sci.* 2006;47(8):3487-3495.

4. Furu L, Onuchic LF, Gharavi A, et al. Milder presentation of recessive polycystic kidney disease requires presence of amino acid substitution mutations. *J Am Soc Nephrol.* 2003;14(8):2004-2014.

5. Losekoot M, Haarloo C, Ruivenkamp C, White SJ, Breuning MH, Peters DJ. Analysis of missense variants in the PKHD1-gene in patients with autosomal recessive polycystic kidney disease (ARPKD). *Hum Genet.* 2005;118(2):185-206.

6. Valente EM, Brancati F, Silhavy JL, et al. AHI1 gene mutations cause specific forms of Joubert syndrome-related disorders. *Ann Neurol.* 2006;59(3):527-534.

7. Bergmann C, Fliegauf M, Brüchle NO, et al. Loss of nephrocystin-3 function can cause embryonic lethality, Meckel-Gruber-like syndrome, situs inversus, and renal-hepatic-pancreatic dysplasia. *Am J Hum Genet.* 2008;82(4):959-970.

8. Bergmann C, Senderek J, Windelen E, et al. Clinical consequences of PKHD1 mutations in 164 patients with autosomal-recessive polycystic kidney disease (ARPKD). *Kidney Int.* 2005;67(3):829-848.

9. Guomin L. QS, Li S., Qi C., Yihui Z., Xiaoyan F., Bingbing W., Hong X. Phenotype analysis of 9 cases with mutations in PKHD1 gene. *Chin J Nephrol.* 2017;33(11):831-837.

10. Wang X, Wang H, Cao M, et al. Whole-exome sequencing identifies ALMS1, IQCB1, CNGA3, and MYO7A mutations in patients with Leber congenital amaurosis. *Human mutation.* 2011;32(12):1450-1459.

11. Onuchic LF, Furu L, Nagasawa Y, et al. PKHD1, the polycystic kidney and hepatic disease 1 gene, encodes a novel large protein containing multiple immunoglobulin-like plexin-transcription-factor domains and parallel beta-helix 1 repeats. *Am J Hum Genet.* 2002;70(5):1305-1317.

12. Otto EA, Schermer B, Obara T, et al. Mutations in INVS encoding inversin cause nephronophthisis type 2, linking renal cystic disease to the function of primary cilia and left-right axis determination. *Nat Genet.* 2003;34(4):413-420.

13. Sayer JA, Otto EA, O'Toole JF, et al. The centrosomal protein nephrocystin-6 is mutated in Joubert syndrome and activates transcription factor ATF4. *Nat Genet.* 2006;38(6):674-681.

14. Reynolds DM, Hayashi T, Cai Y, et al. Aberrant splicing in the PKD2 gene as a cause of polycystic kidney disease. *J Am Soc Nephrol.* 1999;10(11):2342-2351.

15. Ward CJ, Hogan MC, Rossetti S, et al. The gene mutated in autosomal recessive polycystic kidney disease encodes a large, receptor-like protein. *Nat Genet.* 2002;30(3):259-269.

16. Alfares A, Alfadhel M, Wani T, et al. A multicenter clinical exome study in unselected cohorts from a consanguineous population of Saudi Arabia demonstrated a high diagnostic yield. *Mol Genet Metab.* 2017;121(2):91-95.

17. Rossetti S, Consugar MB, Chapman AB, et al. Comprehensive molecular diagnostics in autosomal dominant polycystic kidney disease. *J Am Soc Nephrol.* 2007;18(7):2143-2160.

18. Bielas SL, Silhavy JL, Brancati F, et al. Mutations in INPP5E, encoding inositol polyphosphate-5-phosphatase E, link phosphatidyl inositol signaling to the ciliopathies. *Nat Genet.* 2009;41(9):1032-1036.

19. Katsanis N, Eichers ER, Ansley SJ, et al. BBS4 is a minor contributor to Bardet-Biedl syndrome and may also participate in triallelic inheritance. *Am J Hum Genet.* 2002;71(1):22-29.

20. Hichri H, Stoetzel C, Laurier V, et al. Testing for triallelism: analysis of six BBS genes in a Bardet-Biedl syndrome family cohort. *Eur J Hum Genet.* 2005;13(5):607-616.

21. Taskiran EZ, Korkmaz E, Gucer S, et al. Mutations in ANKS6 cause a nephronophthisis-like phenotype with ESRD. *J Am Soc Nephrol.* 2014;25(8):1653-1661.
