# Supplementary material for: Exome sequencing identifies a likely causative variant in 53% of families with ciliopathy-related features on renal ultrasound after excluding NPHP1 deletions
Source: Genes Dis. 2023 Sep 15;11(5):101111. doi: 10.1016/j.gendis.2023.101111 (PMC11167256; doi:10.1016/j.gendis.2023.101111)
Supplement: Multimedia component 5 [file mmc5.docx]

## Table S4: Detailed information on phenotype and genotype for 9 families with likely causative variants in genes known to cause diseases mimicking the NPHP-RC phenotype (“phenocopies”).

| **Family** | **Gene** | **Disease Entity** | **Zygo-sity** | **Exon** | **Accession No Nt Change AA change dbSNP** | **AA Conser-vation** | **SIFT MT PP2** | **gnomAD** | **ClinVar HGMD ACMG** | **Refe-rence** | **Renal Pheno-type** | **Extra-renal Pheno-type** | **Ethni-city** | **Sex** | **Mbp Homo-zygosity** |
| --- | --- | --- | --- | --- | --- | --- | --- | --- | --- | --- | --- | --- | --- | --- | --- |
| **B3244** | ***COL4A5*** | **Alport-Syndrome** | **het** | **40** | **NM_033380.1 c.3584C>G p.(Pro1195Arg) n/a** | **Xt** | **Tol Dis 0.893** | **n/a** | **- DM at c.3586 and c.3587 LP** | **^1^** | **IE** | **FD, D, GR, CHD, GDD** | **Cauca-sian** | **f** | **8.9** |
| **B3982** | ***COL4A3*** | **Alport-Syndrome** | **het** | **43** | **NM_000091.3 c.3829G>A p.(Gly1277Ser) rs190598500** | **Dm** | **Del Dis 0.999** | **0/102/**  **280750** | **P DM LP** | **^2^** | **C** | **-** | **Arabic** | **m** | **205.6** |
| **B2020** | ***HNF1B*** | **Syndromic CAKUT** | **het** | **4** | **NM_000458.3 c.883C>T p.(Arg295Cys) n/a** | **Ce** | **Del n/a 0.998** | **n/a** | **P DM LP** | **^3^** | **IE, C** | **ID, S** | **Hispanic** | **f** | **9.1** |
| **B2716** | ***HNF1B*** | **Syndromic CAKUT** | **het** | **4** | **NM_000458.3 c.826C>T p.(Arg276*) rs121918672** | **Ns** | **n/a n/a n/a** | **n/a** | **P DM P** | **^4^** | **C** | **-** | **Cauca-sian** | **f** | **6.2** |
| **B3245** | ***PAX2*** | **Syndromic CAKUT** | **het** | **2** | **NM_003990.4 c.56dup p.(Val20Cysfs*34) n/a** | **Fs** | **n/a n/a n/a** | **n/a** | **- DM at c.58 P** | **^5^** | **IE** | **PP** | **Cauca-sian** | **m** | **1.8** |
| **B3979** | ***HNF1B*** | **Syndromic CAKUT** | **het** | **Intron 3** | **NM_000458.3 c.809+1G>A n/a n/a** | **Sp** | **n/a n/a n/a** | **n/a** | **P - P** | **^6^** | **IE, C** | **-** | **Cauca-sian** | **m** | **6.1** |
| **A396** | ***RMND1*** | **COXPD11** | **hom** | **10** | **NM_017909.2 c.1177C>G p.(Leu393Val) n/a** | **Ci** | **Del n/a 0.976** | **n/a** | **- - US** | **Novel** | **IE, SK** | **GDD** | **Turkish** | **f** | **236.7** |
| **B1621** | ***AGXT*** | **NCNL** | **hom** | **5** | **NM_000030.2 c.568G>A p.Gly190Arg rs180177239** | **Dm** | **Tol Dis 0.992** | **0/11/**  **249624** | **LP DM P** | **^7^** | **IE** | **MC, SS, LE** | **Arabic** | **f** | **99.0** |
| **B1821** | ***AGXT*** | **NCNL** | **hom** | **7** | **NM_000030.2 c.731T>C p.(Ile244Thr) rs121908525** | **Dm** | **Del Dis 0.896** | **0/13/**  **282788** | **P DM P** | **^8^** | **IE** | **N** | **Arabic** | **f** | **178.7** |

**Table S4:** Detailed information on phenotype and genotype for 9 families with likely causative variants in genes known to cause diseases mimicking the NPHP-RC phenotype (“phenocopies”). **AA** amino acid; **ACMG** American College of Medical Genetics; **B** benign; **C** cysts; **CAKUT** congenital anomalies of the kidney and urinary tract; **Ce** *Caenorhabditis elegans*; **CHD** congenital heart defect; **Ci** *Ciona intestinalis*; **COXPD11** combined oxidative phosphorylation deficiency 11; **D** deafness; **Del** deleterious; **Dis** disease causing; **DM** disease-causing mutation; **Dm** *Drosophila melanogaster*; **Dr** *Danio rerio*; **f** female; **FD** facial dysmorphism; **Fs** Frameshift; **GDD** global developmental delay; **GR** growth retardation; **het** heterozygous; **HGMD** Human Genome Mutation Database; **hom** homozygous; **ID** intellectual disability; **IE** Increased echogenicity; **LB** likely benign; **LE** low-set ears; **LP** likely pathogenic; **m** male; **Mbp** Megabasepairs; **MC** microcephaly; **MT** Mutation Taster; **N** nystagmus; **NCNL** nephrocalcinosis and nephrolithiasis; **n/a** not available; **Ns** Nonsense; **Nt** Nucleotide; **P** pathogenic; **Poly** polymorphism; **PP** precocious puberty; **PP2** Polyphen2; **S** seizures; **SIFT** Sorting Intolerant From Tolerant; **SK** solitary kidney; **Sp** Splice site; **SS** short stature; **Tol** tolerated; **US** uncertain significance; **Xt** *Xenopus tropicalis*.

**References**

1. Martin P, Heiskari N, Zhou J, et al. High mutation detection rate in the COL4A5 collagen gene in suspected Alport syndrome using PCR and direct DNA sequencing. *J Am Soc Nephrol.* 1998;9(12):2291-2301.

2. Heidet L, Arrondel C, Forestier L, et al. Structure of the human type IV collagen gene COL4A3 and mutations in autosomal Alport syndrome. *J Am Soc Nephrol.* 2001;12(1):97-106.

3. Bellanné-Chantelot C, Clauin S, Chauveau D, et al. Large genomic rearrangements in the hepatocyte nuclear factor-1beta (TCF2) gene are the most frequent cause of maturity-onset diabetes of the young type 5. *Diabetes.* 2005;54(11):3126-3132.

4. Furuta H, Furuta M, Sanke T, et al. Nonsense and missense mutations in the human hepatocyte nuclear factor-1 beta gene (TCF2) and their relation to type 2 diabetes in Japanese. *J Clin Endocrinol Metab.* 2002;87(8):3859-3863.

5. Okumura T, Furuichi K, Higashide T, et al. Association of PAX2 and Other Gene Mutations with the Clinical Manifestations of Renal Coloboma Syndrome. *PLoS One.* 2015;10(11):e0142843.

6. Heidet L, Decramer S, Pawtowski A, et al. Spectrum of HNF1B mutations in a large cohort of patients who harbor renal diseases. *Clin J Am Soc Nephrol.* 2010;5(6):1079-1090.

7. Talati JJ, Hulton SA, Garrelfs SF, et al. Primary hyperoxaluria in populations of Pakistan origin: results from a literature review and two major registries. *Urolithiasis.* 2018;46(2):187-195.

8. von Schnakenburg C, Rumsby G. Primary hyperoxaluria type 1: a cluster of new mutations in exon 7 of the AGXT gene. *J Med Genet.* 1997;34(6):489-492.
