# Supplementary material for: Exome sequencing identifies a likely causative variant in 53% of families with ciliopathy-related features on renal ultrasound after excluding NPHP1 deletions
Source: Genes Dis. 2023 Sep 15;11(5):101111. doi: 10.1016/j.gendis.2023.101111 (PMC11167256; doi:10.1016/j.gendis.2023.101111)
Supplement: Multimedia component 6 [file mmc6.docx]

## Table S5: Detailed information on phenotype and genotype for 13 families with likely causative variants in potential novel monogenic disease candidate genes.

| **Family** | **Genes** | **Zygo-sity** | **Exon** | **Accession No Nt Change AA change dbSNP** | **AA Conser-vation** | **SIFT MT PP2** | **gnomAD** | **ClinVar HGMD** | **Refe-rence** | **Renal Pheno-type** | **Extra-renal Pheno-type** | **Ethnicity** | **Sex** | **Mbp Homo-zygosity** |
| --- | --- | --- | --- | --- | --- | --- | --- | --- | --- | --- | --- | --- | --- | --- |
| **A5277** | ***NHSL2*** | **hom** | **6** | **NM_001013627.2 c.1718G>A p.(Arg573His) n/a** | **Dr** | **Del Dis 0.947** | **n/a** | **- -** | **Novel** | **C, IE** | **-** | **Cauca-sian** | **m** | **5.9** |
| **B1700** | ***NTN5*** | **hom** | **3** | **NM_145807.2 c.727C>T p.(Arg243Cys) rs376906018** | **Sc** | **Del Dis 0.995** | **1/95/**  **274778** | **- -** | **Novel** | **C** | **-** | **Vietna-mese** | **f** | **15.4** |
| **B2014 (AS)** | ***TNS1*** | **het** | **24** | **NM_022648.5 c.3593G>A p.(Arg1198Gln) rs369393471** | **Dr** | **Tol n/a 0.903** | **0/186/**  **251068** | **- -** | **Novel** | **ESRD** | **SS, T** | **Guate-malan** | **m** | **32.1** |
|  |  | **het** | **18** | **NM_022648.5 c.2818C>T p.(Arg940Trp) rs776215061** | **Ci** | **Del n/a 0.999** | **0/9/**  **281906** | **- -** | **Novel** |  |  |  |  |  |
| **B2014 (IP)** | ***TNS1*** | **het** | **24** | **NM_022648.5 c.3593G>A p.(Arg1198Gln) rs369393471** | **Dr** | **Tol n/a 0.903** | **0/186/**  **251068** | **- -** | **Novel** | **IE, PU** | **SS, T** | **Guate-malan** | **m** | **37.5** |
|  |  | **het** | **18** | **NM_022648.5 c.2818C>T p.(Arg940Trp) rs776215061** | **Ci** | **Del n/a 0.999** | **0/9/**  **281906** | **- -** | **Novel** |  |  |  |  |  |
| **B2033** | ***TFCP2L1*** | **hom** | **9** | **NM_014553.2 c.869del p.(Ser290Phefs*50) n/a** | **Fs** | **n/a n/a n/a** | **n/a** | **- -** | **Novel** | **IE** | **LCA, N** | **Arabic** | **f** | **137.2** |
| **B2283** | ***IQCA1*** | **het** | **17** | **NM_001270585.1 c.1993C>T p.(Arg665Cys) rs186626813** | **Sc** | **Del Dis 1** | **1/196/**  **278770** | **- -** | **Novel** | **C** | **-** | **Malay-sian** | **m** | **39.7** |
|  |  | **het** | **2** | **NM_001270585.1 c.227G>A p.(Arg76Gln) rs372732894** | **Dm** | **Del Dis 0.621** | **0/43/**  **280468** | **- -** | **Novel** |  |  |  |  |  |
| **B2435** | ***GPRC6A*** | **hom** | **4** | **NM_148963.4 c.1391T>A p.(Phe464Tyr) rs143913245** | **Dm** | **Del Dis 1** | **3/418/**  **282060** | **- -** | **Novel** | **IE** | **RP** | **Malay-sian** | **f** | **18.9** |
| **B2563** | ***DEGS1*** | **hom** | **2** | **NM_003676.3 c.99A>G p.(Ile33Met) rs780472867** | **Dm** | **Del Dis 0.955** | **0/8/**  **238444** | **- -^[[1]](#footnote-1)^** | **^1^** | **IE, LCD** | **-** | **Arabic** | **f** | **#REF!** |
| **B3117** | ***RHPN1*** | **hom** | **8** | **NM_052924.2 c.940G>A p.(Ala314Thr) rs199609984** | **Sc** | **Tol Dis 0.385** | **1/431/**  **184712** | **- -** | **Novel** | **IE** | **GR** | **Arabic** | **f** | **#REF!** |
| **B3169** | ***TRPM4*** | **het** | **9** | **NM_017636.4 c.1082T>G p.(Leu361Arg) rs148763371** | **Ce** | **Del Dis 0.929** | **0/82/**  **282800** | **US -^[[2]](#footnote-2)^** | **^2,3^** | **IE** | **-** | **Cauca-sian** | **m** | **17.0** |
|  |  | **het** | **18** | **NM_017636.4 c.2665del p.(His889Thrfs*35) rs777047595** | **Fs** | **- - -** | **0/38/**  **282644** | **US -^[[3]](#footnote-3)^** | **^2,3^** |  |  |  |  |  |
| **B3632** | ***GLOD5*** | **hemi** | **4** | **NM_001080489.2 c.437A>G p.(Asp146Gly) n/a** | **Ce** | **Del Dis 1** | **0/1/1/**  **180647** | **- -** | **Novel** | **IE** | **PCT, IH, LMT** | **Cauca-sian** | **m** | **5.2** |
| **B3931** | ***GPR63*** | **hom** | **3** | **NM_001143957.2 c.1252G>A p.(Val418Met) rs372859048** | **Dr** | **Del Dis 0.907** | **0/81/**  **282118** | **- -** | **Novel** | **C** | **DC** | **Kuala Lumpur** | **f** | **8.2** |
| **B3957** | ***ZNF679*** | **het** | **5** | **NM_153363.2 c.733T>C p.(Cys245Arg) rs34967493** | **Ce** | **Del Dis 0.998** | **5/1130/**  **280030** | **- -** | **Novel** | **IE, LCD, PU** | **HSM, HF** | **Cauca-sian** | **m** | **18.4** |
|  |  | **het** | **5** | **NM_153363.2 c.1220C>T p.(Pro407Leu) rs1439899398** | **Ci** | **Tol Dis 0.01** | **0/3/**  **224104** | **- -** | **Novel** |  |  |  |  |  |
| **B4026** | ***MEAK7*** | **hom** | **5** | **NM_020947.4 c.849_850del p.(Cys283Trpfs*17) rs778301698** | **Fs** | **n/a n/a n/a** | **0/5/**  **251094** | **- -** | **Novel** | **C** | **AP** | **Cauca-sian** | **f** | **5.7** |

**Table S5:** Detailed information on phenotype and genotype for 13 families with likely causative variants in potential novel monogenic disease candidate genes. **AA** amino acid; **AP** abdominal pain; **C** cysts; **Ce** *Caenorhabditis elegans*; **Ci** *Ciona intestinalis*; **DC** dilatative cardiomyopathy; **Del** deleterious; **Dis** disease causing; **Dm** *Drosophila melanogaster*; **Dr** *Danio rerio*; **ESRD** end stage renal disease; **f** female; **Fs** Frameshift; **GR** growth retardation; **hemi** hemizygous; **het** heterozygous; **HF** hepatic fibrosis; **HGMD** Human Genome Mutation Database; **hom** homozygous; **HSM** hepatosplenomegaly; **IE** Increased echogenicity; **IH** intraventricular hemorrhage; **LCA** Leber congenital amaurosis; **LCD** loss of corticomedullary differentiation; **LMT** low muscle tone; **m** male; **Mbp** Megabasepairs; **MT** Mutation Taster; **N** nystagmus; **n/a** not available; **Nt** Nucleotide; **PCT** polycytemia; **PP2** Polyphen2; **PU** proteinuria; **RP** retinitis pigmentosa; **Sc** *Saccharomyces cerevisiae*; **SIFT** Sorting Intolerant From Tolerant; **SS** short stature; **T** tachypnea; **Tol** tolerated; **US** uncertain significance; **Xt** *Xenopus tropicalis*.

**References**

1. Pant DC, Dorboz I, Schluter A, et al. Loss of the sphingolipid desaturase DEGS1 causes hypomyelinating leukodystrophy. *J Clin Invest.* 2019;129(3):1240-1256.

2. Kruse M, Schulze-Bahr E, Corfield V, et al. Impaired endocytosis of the ion channel TRPM4 is associated with human progressive familial heart block type I. *J Clin Invest.* 2009;119(9):2737-2744.

3. Wang H, Xu Z, Lee BH, et al. Gain-of-Function Mutations in TRPM4 Activation Gate Cause Progressive Symmetric Erythrokeratodermia. *J Invest Dermatol.* 2019;139(5):1089-1097.

1. Gene previously published as disease-causing in hypomyelinating leukodystrophy if mutated [↑](#footnote-ref-1)
2. Gene previously published as disease-causing in cardiac conduction disease and erythrokeratodermia if mutated [↑](#footnote-ref-2)
3. Gene previously published as disease-causing in cardiac conduction disease and erythrokeratodermia if mutated [↑](#footnote-ref-3)
