# Supplementary material for: Exome sequencing identifies a likely causative variant in 53% of families with ciliopathy-related features on renal ultrasound after excluding NPHP1 deletions
Source: Genes Dis. 2023 Sep 15;11(5):101111. doi: 10.1016/j.gendis.2023.101111 (PMC11167256; doi:10.1016/j.gendis.2023.101111)
Supplement: Multimedia component 7 [file mmc7.docx]

## Table S6. Detailed information on phenotype for 35 families without likely causative variants.

| **Family** | **Renal Phenotype** | **Extrarenal Phenotype** | **Ethnicity** | **Sex** | **Mbp Homozygosity** |
| --- | --- | --- | --- | --- | --- |
| **A47** | **C, IE** | **-** | **Caucasian** | **m** | **20.9** |
| **B1697** | **C** | **-** | **Vietnamese** | **f** | **22.2** |
| **B1698** | **C** | **-** | **Vietnamese** | **m** | **32.3** |
| **B1699** | **C** | **-** | **Vietnamese** | **m** | **13.7** |
| **B1713** | **C** | **-** | **Caucasian** | **f** | **18.8** |
| **B2000**  **(IP)** | **C** | **FD, MC, RP, ID, SS, SDC** | **Egyptian** | **f** | **91.0** |
| **B2000**  **(AS)** | **-** | **GDD, MC, FD, SDC, HSM** | **Egyptian** | **f** | **91.0** |
| **B2024** | **IE** | **-** | **Caucasian** | **m** | **52.1** |
| **B2304** | **IE** | **SS, T, CHD** | **Guatemalan** | **m** | **16.2** |
| **B2406** | **IE** | **H, CHD** | **Caucasian** | **m** | **4.8** |
| **B2436** | **IE, C, LCD** | **-** | **Egyptian** | **f** | **276.7** |
| **B2438** | **C** | **-** | **Egyptian** | **m** | **9.8** |
| **B2545** | **IE** | **-** | **Egyptian** | **f** | **4.2** |
| **B2552** | **IE** | **CH** | **Egyptian** | **m** | **166.5** |
| **B2597** | **IE** | **SS** | **Pakistani** | **m** | **17.7** |
| **B2601** | **IE, LCD** | **SS** | **Pakistani** | **m** | **5.8** |
| **B2656** | **IE** | **-** | **Caucasian** | **m** | **5.1** |
| **B2714** | **IE** | **SS** | **Caucasian** | **f** | **9.2** |
| **B2858** | **C** | **-** | **Pakistani** | **m** | **113.5** |
| **B3054** | **IE** | **-** | **Caucasian** | **f** | **27.6** |
| **B3238**  **(IP)** | **IE** | **-** | **Arabic** | **f** | **150.9** |
| **B3238**  **(AS)** | **IE, C** | **-** | **Arabic** | **m** | **155.4** |
| **B3273** | **C** | **-** | **Arabic** | **m** | **15.0** |
| **B3275** | **C** | **-** | **Arabic** | **m** | **19.8** |
| **B3303** | **C** | **-** | **Caucasian** | **f** | **2.3** |
| **B3542** | **IE** | **-** | **Caucasian** | **f** | **11.6** |
| **B3595** | **C** | **-** | **Caucasian** | **m** | **10.9** |
| **B3599** | **C** | **-** | **Caucasian** | **f** | **3.2** |
| **B3631** | **C** | **MC** | **Arabic** | **f** | **116.3** |
| **B3633** | **IE** | **RP** | **Caucasian** | **m** | **7.8** |
| **B3695** | **IE, LCD** | **SS** | **Pakistani** | **f** | **213.3** |
| **B3810** | **C** | **GDD, LMT** | **Caucasian** | **m** | **5.3** |
| **B3880** | **IE** | **-** | **Arabic** | **f** | **14.9** |
| **B3930** | **IE, LCD** | **POD** | **Kuala Lumpur** | **m** | **15.1** |
| **B2525** | **C** | **-** | **Caucasian** | **f** | **6.0** |
| **B2599** | **IE, C** | **SS** | **Arabic** | **f** | **82.3** |
| **B3977** | **IE, C** | **-** | **Caucasian** | **f** | **7.8** |

**Table S6:** Detailed information on phenotype for 32 families without likely causative variants. **C** cysts; **CH** cerebellar hypoplasia; **CHD** congenital heart defect; **f** female; **FD** facial dysmorphism; **GDD** global developmental delay; **H** hydrocephalus; **HSM** hepatosplenomegaly; **ID** intellectual disability; **IE** Increased echogenicity; **LCD** loss of corticomedullary differentiation; **LMT** low muscle tone; **m** male; **Mbp** Megabasepairs; **MC** microcephaly; **PCT** polycytemia; **POD** pale optic discs; **RP** retinitis pigmentosa; **SDC** syndactyly; **SS** short stature; **T** tachypnea.
